# Root Development and Stress Tolerance in rice: The Key to Improving Stress Tolerance without Yield Penalties

**DOI:** 10.3390/ijms21051807

**Published:** 2020-03-06

**Authors:** Deok Hyun Seo, Subhin Seomun, Yang Do Choi, Geupil Jang

**Affiliations:** 1 School of Biological Sciences and Technology, Chonnam National University, Gwangju 61186, Korea; 198478@jnu.ac.kr (D.H.S.); 171965@jnu.ac.kr (S.S.); 2 The National Academy of Sciences, Seoul 06579, Korea; choiyngd@snu.ac.kr

**Keywords:** root, auxin, abiotic stress, tolerance, rice

## Abstract

Roots anchor plants and take up water and nutrients from the soil; therefore, root development strongly affects plant growth and productivity. Moreover, increasing evidence indicates that root development is deeply involved in plant tolerance to abiotic stresses such as drought and salinity. These findings suggest that modulating root growth and development provides a potentially useful approach to improve plant abiotic stress tolerance. Such targeted approaches may avoid the yield penalties that result from growth–defense trade-offs produced by global induction of defenses against abiotic stresses. This review summarizes the developmental mechanisms underlying root development and discusses recent studies about modulation of root growth and stress tolerance in rice.

## 1. Introduction

Abiotic stress, including drought, low or high temperature, and salinity, seriously affects growth and productivity of plants [1]. Furthermore, global climate change may be increasing the frequency and severity of abiotic stress, thus threatening food production around the world [2]. In nature, plants are continuously exposed to different abiotic stresses and have evolved a variety of defense mechanisms to survive these abiotic stresses. Therefore, manipulation of these defenses may allow us to produce crop plants with improved tolerance to abiotic stress [3]. To understand the mechanisms of these stress responses and enable approaches to improve stress tolerance, extensive studies have identified many genetic components that mediate plant responses to abiotic stresses. These studies further revealed that abscisic acid (ABA) and jasmonic acid (JA) are key signaling molecules that mediate plant responses to abiotic stresses [4,5,6,7].

The essential roles of ABA and JA in plant stress responses and stress tolerance provide important clues for the development of stress-tolerant plants, and many successful studies reported stress-tolerant crops generated by modulation of ABA or JA responses. For example, activation or overexpression of *OsPYL10*, an ABA receptor increased ABA responses, leading to improved tolerance to drought [8], and overexpression of *OsbZIP42* or heterologous expression of *ZmbZIP4* (encoding positive regulators of ABA signaling) conferred drought tolerance in rice [9,10]. Additionally, a knock-out of the JA signaling repressor *OsJAZ1* increased JA sensitivity and drought tolerance; conversely, overexpression of *OsJAZ1* reduced JA sensitivity and drought tolerance [11]. These studies suggested that modulation of ABA or JA responses is a key strategy to enhance tolerance to abiotic stress. However, because of growth–defense trade-offs, activation of defense systems frequently reduces growth and productivity in plants [12]. Indeed, a growing number of studies reported that enhancing ABA or JA responses negatively affects plant growth and productivity under normal growth conditions [13,14,15].

Targeting specific tissues or organs, rather than altering responses on a whole-plant basis, has emerged as an alternative strategy for the development of crops with improved abiotic stress tolerance and growth. The root system is a key target for this strategy, as roots are responsible for the uptake of water and nutrients, as well as plant anchorage in soil; therefore, plant fitness and productivity largely depend on root development. Based on the potential for root development to influence crop production, optimization of root development is expected to be crucial for enabling the next Green Revolution [16]. In addition, root development affects the plant response to environmental conditions, and developmental plasticity of roots may help plants to survive abiotic stress conditions [17,18]. More importantly, several studies reported that modulating root development improved stress tolerance in crops and increased yield [19,20,21,22]. These observations suggested that modulation of root development could be a key strategy for development of crops with improved stress tolerance and yield, minimizing or avoiding the penalties of growth–defense trade-offs. 

Rice is an important staple crop supporting approximately two-thirds of the world’s population [23]. The rice root system is composed of seminal, crown, and lateral roots. The seminal root develops from the seed during embryogenesis, and the crown roots that constitute the major root system of rice develop from the stem during post-embryogenesis. Lateral roots develop from lateral root primordia that originate from pericycle cells of seminal and crown roots, and the formation of lateral roots is responsible for extensive increase in the surface area of the root system for an uptake of water and nutrient from the soil [24,25]. The molecular and genetic mechanisms underlying root development in rice remain less well-studied than those of *Arabidopsis*. However, increasing evidence suggests that rice and *Arabidopsis* share many mechanisms of root development, including the function of auxin as a key regulator, and modulation of root development is a good strategy to improve stress tolerance without yield penalties in rice. In this review, we briefly describe the mechanisms underlying root development in *Arabidopsis* and rice, focusing on auxin. Additionally, we discuss recent studies reporting enhanced abiotic stress tolerance by modulating root development in rice.

## 2. Root Development in *Arabidopsis*

### 2.1. Root Formation and Growth 

Roots initially form during embryogenesis through cell division of the zygote. The zygote asymmetrically divides to form two cells: a small cell in the apical position and a large cell in the basal position. The small cell divides vertically to form the proembryo and the large cell divides horizontally to form the suspensor, which connects the embryo to the maternal tissue. The proembryo contributes the stem cells for root epidermis, ground, and vascular tissues and the uppermost cell of the suspensor develops into the hypophysis, a progenitor of the quiescent center (QC), which is responsible for determination of cell identity and meristematic activity of root stem cells by preventing differentiation of the stem cells [26,27,28]. The embryonic root continues to grow in the apical direction, and the growth is promoted by the division of stem cells in the root apical meristem (RAM). The RAM is composed of undifferentiated cells and plays a central role in the growth of roots by protecting the stem cell niche and maintaining the cell division activity of the undifferentiated cells. Therefore, RAM activity required for root development is regulated by balancing cell division and differentiation, and auxin mediates this process [29,30]. 

### 2.2. Auxin and Root Development

**Figure 1:**
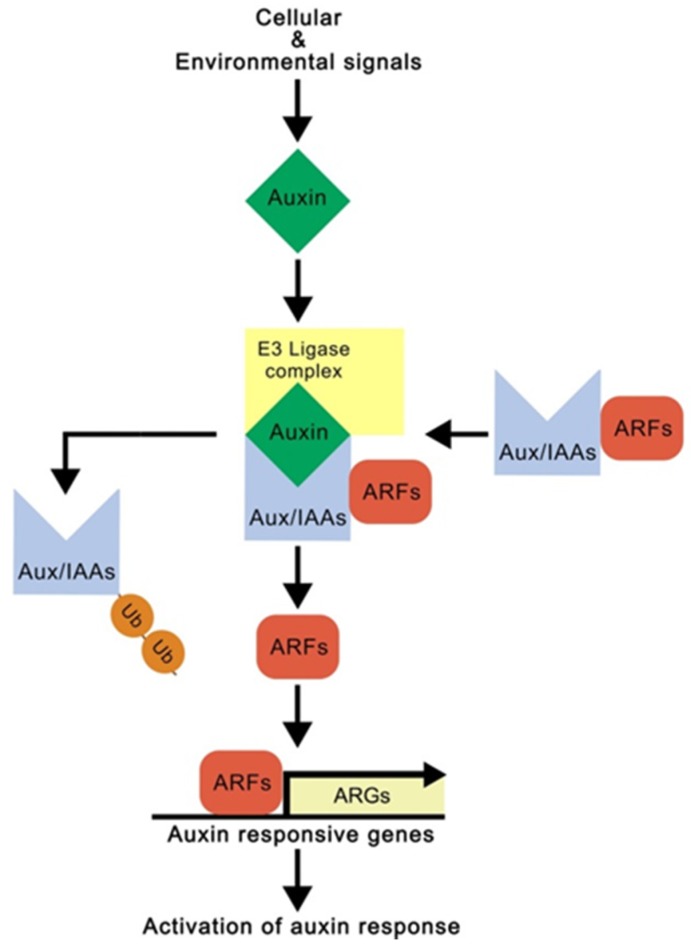
A schematic of the auxin signaling pathway. ARFs and Aux/IAAs function as positive and negative regulators in the auxin signaling pathway. In response to cellular and environmental signals, auxin is biosynthesized and the auxin promotes E3 ligase complex-mediated proteolysis of Aux/IAAs. The degradation leads to the release of ARFs and the activation of auxin response. Ub and ARGs indicate ubiquitin and auxin-responsive genes, respectively.

Auxin is a key regulator of plant development and growth, determining cell identity and regulating cell division and differentiation. Previously many studies demonstrated that regulation of auxin response is a key process controlling root development [31,32,33]. Auxin biosynthesis and signaling pathway are deeply involved in the auxin-mediated root development, and the results showing that root development is strongly inhibited in auxin signaling or deficiency mutants such as *axr3-1* and *trp2-12* support this [34,35]. In plants, indole-3-acetic acid is the predominant natural auxin and is biosynthesized through tryptophan-independent and -dependent pathways [36,37]. The tryptophan-dependent pathway is mediated by tryptophan aminotransferase, which converts tryptophan to indole-3-pyruvate, and by flavin monooxygenase, which converts indole-3-pyruvate to auxin. This tryptophan-dependent pathway is currently the best understood auxin biosynthetic pathway in plants. The biosynthesis of auxin induces auxin responses through the auxin signaling pathway, which mediates E3 ligase complex-mediated proteolysis of Aux/IAA auxin signaling repressor proteins (Figure 1). The proteolysis of Aux/IAAs leads to the release of AUXIN RESPONSE FACTORs (ARFs), which activate transcription of auxin-responsive genes [38,39]. ARFs such as MONOPTEROS (MP/ARF5) and NONPHOTOTROPIC HYPOCOTYL4 (NPH4/ARF4) initially mediate root responses to auxin, and these ARFs, in turn, activate transcriptional expression of auxin-responsive transcription factor *PLETHORAs* (*PLTs*), which play a pivotal role in maintenance of the stem cell niche and cell proliferation in a dosage-dependent manner [40,41,42]. 

In roots, auxin accumulates at the root tip, specifically in the root stem cells. These cells specifically express auxin biosynthesis genes [43,44]. In addition to the root tip-specific expression of auxin biosynthesis genes, polar auxin transport is largely responsible for root tip-specific accumulation of auxin. This transport is mediated by polar auxin transporters such as PIN-FORMED PROTEINs (PINs) and AUXIN TRANSPORTER PROTEINs (AUXs) P-GLYCOPROTEINs (PGPs) [45,46]. Polar auxin transport generates an auxin gradient in the RAM, with high auxin at the proximal position and low auxin at the distal position of the RAM [47]. The auxin gradient is essential for maintenance of RAM activity and root development, and previous studies showing that mutation of genes involved in polar auxin transport inhibits root development by disrupting proper distribution of auxin in roots, suggest that polar auxin transport is another key process controlling the tissue-specific auxin required for root development [48,49,50].

Root growth is strongly affected by abiotic stresses such as drought and salinity, and auxin is deeply involved in this process. Root growth is suppressed in response to the stress hormone JA, and a study by Chen et al. (2011) suggested that auxin-responsive *PLTs* mediate this suppression [51]. Indeed, exogenous JA treatment reduces the expression of *PLTs* in wild-type plants but not in JA-signaling mutants such as *coi1-1* and *myc2*. Furthermore, the JA response factor MYC2, which induces expression of JA-responsive genes, directly binds to the promoter of *PLTs,* and suppresses the expression of *PLTs*. These findings indicated that MYC2 mediates JA-induced inhibition of apical root growth by directly suppressing the expression of auxin-responsive *PLTs.* This also suggested that auxin and its downstream regulators mediate the stress-induced inhibition of root growth, and the hypothesis is supported by an increasing number of studies [52,53,54]. 

Root growth is also regulated by other developmental processes including cell wall development. Plant cells are encased by cell walls mostly made of three types of polysaccharides: cellulose, hemicelluloses, and pectins, and cortical microtubules regulate development of cell walls by guiding cellulose deposition [55,56]. Plant cell walls are flexible and diverse, and cell wall development is involved in the growth of roots because roots are composed of a variety type of cells with different functions and developmental stages [55]. Indeed, *bul, bot1,* and *prc1* mutants, which have defects in microtubule organization and cellulose synthesis, exhibit short-root phenotypes [57,58,59]. The relationship between auxin and cell wall development remains largely unknown in root development, but a growing number of studies propose that auxin is possibly involved in the cell wall-mediated root development [60,61]. 

## 3. Rice Root Development

**Figure 2:**
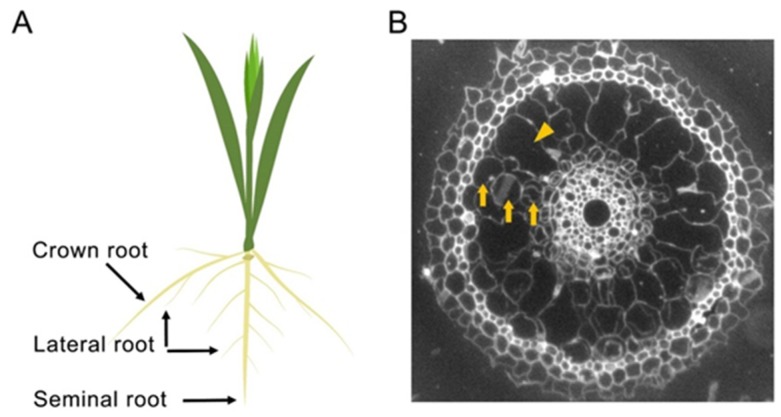
Anatomy of rice roots. (**A**) A schematic of rice root system composed of seminal, crown and lateral roots. (**B**) A radial anatomy of rice roots was visualized by transverse sectioning of 12-day-old seminal roots. Arrow and arrowhead indicate cortex and aerenchyma, respectively.

Rice roots have several distinct characteristics compared to *Arabidopsis* roots. In dicots, including *Arabidopsis*, roots can grow laterally via cell proliferation in the cambium layer, which increases the diameter of the root [62]. Cambium develops from procambium, and has higher meristematic activity. In contrast to dicot plants, the procambium of monocots differentiates into vascular tissues and consequently, cambium does not form and lateral growth does not occur [62]. In addition, *Arabidopsis* roots form a single layer of cortex between epidermis and endodermis, but rice roots form a multi-layer cortex by periclinal division of endodermal cells [63,64] (Figure 2). During maturation, the multi-layer cortex is destroyed by programmed cell death and develops into aerenchyma, a gas space that facilitates internal oxygen transport [65]. The precise developmental and molecular mechanisms that are specific to rice root development largely remain unknown. However, ongoing studies revealed that overall, the rice root system shares similar mechanisms to those of *Arabidopsis*, and that auxin is a key regulator of rice root development.

### 3.1. Formation of Crown Roots in Rice

Although the seminal root is the first to emerge from the seed, crown roots constitute the major root system of rice. Crown roots develop from a meristem on the lowest node of the stem; this radial meristem shares common characteristics with the root pericycle tissues responsible for the formation of lateral roots [66,67]. The first identified gene related to crown root formation was *CROWN ROOTLESS1/ADVENTITIOUS ROOTLESS1* (*CRL1*) [68]. *CRL1* is specifically expressed in the tissues where crown roots are initiated and auxin promotes *CRL1* expression. Indeed, *CRL1* has auxin response elements in its promoter and is a direct target of ARF. In the *crl1* mutant, crown root formation is strongly inhibited. Together, these observations indicated that *CRL1* regulates the formation of crown roots, and auxin signaling is deeply involved in this process. Studies using auxin biosynthesis and signaling mutants further demonstrated the role of auxin in crown root formation. Transgenic rice overexpressing the auxin biosynthetic gene *OsYUCCA1* formed more crown roots, whereas a gain-of-function mutant of *OsIAA23* developed fewer crown roots [69,70]. Polar auxin transport also mediates the auxin-dependent crown root formation. *OsPIN1* responsible for auxin distribution is specifically expressed in lateral root primordia of crown roots. A knock-down of *OsPIN1* by RNA interference suppressed crown root formation, and treatment of NPA (N-1-naphthylphalamic acid, an auxin-transport inhibitor) mimicked the knock-down effect of *OsPIN1* on crown root formation [71]. A study of *OsGNOM1* also supports the essential role of polar auxin transport in crown root formation. *GNOM* encodes a large guanine nucleotide exchange factor for the ADP-ribosylation factor, and regulates auxin transport in *Arabidopsis* [72]. In rice, *OsGNOM1* regulates expression of auxin efflux genes such as *OsPIN2*, *OsPIN5b*, and *OsPIN9*, and the *osgnom1* mutant has defects in crown root formation [73]. In addition, identification and characterization of regulators such as *CRL6* and *WOX11* further support the role of auxin in crown root formation in rice. Auxin rescued the defective crown root formation in *crl6* mutant, and endogenous auxin accumulation by *OsYUCCA1* overexpression or exogenous auxin treatment promoted expression of *WOX11*, a key positive regulator of crown root formation [74,75,76]. A recent study using *OsSPL3* revealed that microRNA signaling is involved in formation of crown roots in rice, and suggested that *OsmiR156*-*OsSPL3* module regulates crown root formation by controlling auxin signaling [77].

### 3.2. Formation of Lateral Roots in Rice

Similar to the formation of crown roots, the formation of lateral roots largely depends on auxin and auxin-related genetic components. Formation of lateral roots extensively increases the surface area of root systems for resource acquisition from the soil [24,25]. Lateral roots originate primarily from pericycle tissue of roots, and early studies using *altered lateral root formation 1* (*alf1*) and *lateral rootless 1 (lrt1)* suggested that auxin is involved in lateral root formation in rice [78,79]. A mutant rice *arm1* isolated by screening for resistance to 2,4-dichlorophenoxyacetic acid (2,4-D) was defective in lateral root formation [78]. *lrt1* mutant rice has defects in the formation of lateral roots, and the lateral root-less phenotype of *lrt1* mutants was rescued by exogenous auxin [79], suggesting that auxin is involved in this process.

The involvement of auxin in lateral root formation has been further demonstrated by several studies. For example, mutation of *OsAUX1* (encoding an auxin influx carrier) suppressed the lateral root initiation, while overexpression of *OsAUX1* promoted lateral root formation [80]. RNAi silencing of *OsMADS25* (encoding a transcription factor that directly represses *OsIAA14* expression) reduced lateral root formation as well as root elongation by controlling auxin responses [81,82]. Although accumulating evidence indicates that auxin is a key regulator of lateral root formation, the precise molecular mechanism underlying this process remains largely unelucidated in rice.

### 3.3. Root Growth in Rice

**Figure 3:**
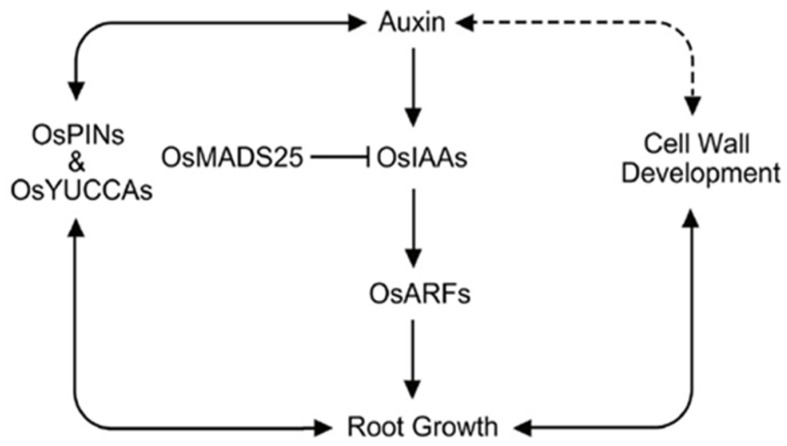
A schematic of auxin-mediated root growth in rice. Auxin response regulates root growth in rice, and the auxin response is controlled by auxin-related genes such as *OsYUCCAs* for auxin biosynthesis, *OsPINs* for transport, and *OsARFs* for signaling. OsMADS25 is not a direct component of the auxin signaling pathway, but regulates root growth by controlling the expression of *OsIAAs*. Cell wall development also regulates root growth in rice, and it is likely that auxin is involved in this process. Dashed arrow indicates hypothetical regulation.

Since meristematic activity of the RAM is essential for the growth of roots in rice, mutant rice with defects in division and survival of RAM cells show severe defects in root growth [83]. Similar to the root system of *Arabidopsis*, auxin responses controlled by auxin biosynthesis, polar auxin transport, and signaling pathways are key factors determining RAM activity and the growth of roots (Figure 3). ARFs play an essential role in the regulation of auxin responses by governing transcription of auxin-responsive genes. In rice, *OsARF12* controls apical root growth by regulating the expression of *OsYUCCAs* for auxin biosynthesis and *OsPINs* for polar auxin transport [84]. Consequently, mutant plants that lack expression of *OsARF12* showed lower auxin levels in their roots and formed shorter roots, compared to wild-type plants. This indicates that, in addition to affecting root initiation, auxin affects root growth in rice.

The essential role of auxin in the growth of rice roots is further supported by recent studies using *OsPIN1b* and *OsMADS25* [81,82]. A study of *OsPIN1b* (encoding an auxin efflux carrier) showed that *OsPIN1b* is specifically expressed in the rice root tip and mutant plants that lack expression of *OsPIN1b* developed short roots [81]. Unlike ARFs and IAAs, the OsMAD25 transcription factor is not a direct component of the auxin signaling pathway. However, OsMAD25 represses transcription of *OsIAA14* (encoding a repressor of auxin signaling) by directly binding to the CArG-box in the promoter of *OsIAA14*. As expected, overexpression of *OsMAD25* promoted rice root growth, whereas RNAi-mediated silencing of *OsMAD25* reduced root growth, supporting the essential role of auxin in growth of rice roots [82].

Iron homeostasis is involved in the plant response to stress [85] and a study of *OsARF12* suggested a relationship between root growth and stress responses [84]. This study showed that *osarf12* mutant rice with defects in auxin signaling formed short roots compared with wild-type plants and affected iron homeostasis by reducing the expression of *MITOCHONDRIAL IRON-REGULATED* (*OsMIR*) and inducing expression of *SHORT POSTEMBRYONIC ROOT1* (*OsSPR1*). Previously, it was shown that *OsMIR* and *OsSPR1* positively and negatively regulate iron homeostasis, respectively, and consequently, knockout of *OsMIR* and *OsSPR1* affects iron homeostasis [86,87]. These results support that *OsARF12* responsible for auxin-mediated root growth also regulates iron homeostasis by controlling the expression of *OsMIR* and *OsSPR1*. Although the precise relationship between root growth and iron homeostasis remains elusive, this finding suggests that auxin-mediated root growth might be involved in iron homeostasis and plant stress responses.

Similar to the development of *Arabidopsis* roots, development of cell walls is involved in the growth of rice roots. In rice, *OsGLU3* encodes a membrane-bound endo-1,4-β-glucanase acting on β-glucans, a major polysaccharide component of rice cell walls [88,89]. *OsGLU3* is predominately expressed in roots, and *OsGLU3* proteins localize at the plasma membrane. *Osglu3* mutant rice exhibited lower crystalline cellulose contents in root cell walls. The RAM of the *Osglu3* mutant is smaller than that of wild-type plants, leading to a short-root phenotype. This suggested that development of root cell walls modulates the growth of roots, and a study showing that knock-down of the *OsEXPA8* (encoding an enzyme responsible for cell wall development) induced short root phenotype supported this [90]. The smaller size of the RAM in the cell wall mutants indicated that the short-root phenotype was caused by the reduced RAM activity. Since auxin is a key regulator of RAM activity, these observations suggested that auxin is involved in cell wall-mediated root growth (Figure 3), and a study using *OsMOGS* (encoding mannosyl-oligosaccharide glucosidase for cell wall formation) partially supported this [91]. In this study, *Osmogs* mutant rice with low auxin contents had impaired root growth and reduced cell wall thickness due to decreased cellulose synthase.

### 3.4. Complexity of Auxin-Mediated Root Growth and Development

Auxin is a key regulator promoting root formation and growth in rice. However, root phenotypes of several mutants suggest that the developmental mechanisms controlling root formation and growth downstream auxin are not simple. For example, rice *auxin resistant mutant 1* (*arm1*) does not respond to auxin, and forms longer roots. Interestingly, the mutant develops fewer lateral roots than wild-type plants [78]. Overexpression of *OsYUCCA1* (encoding an enzyme responsible for auxin biosynthesis) promotes formation of crown roots, but suppresses root growth [69]. In addition, suppression of *OsESG1* (encoding an S-domain receptor-like kinase) promotes root elongation, but reduces the formation of crown roots by regulating auxin distribution [92]. Unlike *ARM1*, *OsYUCCA1,* and *OsESG1*, *OsMADS25* promotes root growth and lateral root formation at the same time. Both root growth and lateral root formation was suppressed by RNAi silencing of *OsMADS25*, but was promoted by overexpression of *OsMADS25* [82]. These findings suggest that auxin pathways responsible for root formation and growth interact in a complex manner, and the complexity may be involved in developmental flexibility of root development in response to ever-changing environmental conditions, including abiotic stress.

## 4. Root Development and Stress Tolerance in Rice

Previous studies of the relationship between root development and stress tolerance suggested that root development affects plant tolerance to abiotic stresses, including drought [93,94,95]. Several studies of OsNAC transcription factors have convincingly shown the role of roots in stress tolerance. NAC transcription factors are plant-specific transcription factors involved in development and stress responses [96,97]. Whole-plant expression of drought-responsive *OsNAC5*, *OsNAC6*, *OsNAC9*, and *OsNAC10* increased drought tolerance by promoting root growth in width [19,20,21,22]. More importantly, root-specific overexpression of these transcription factors promoted the root diameter and stress tolerance more effectively than whole-body overexpression. The improved tolerance led to an increase in rice yield under drought conditions. For example, transgenic rice with root-specific overexpression of *OsNAC5*, *OsNAC6*, *OsNAC9*, and *OsNAC10* showed 22–63%, 27–74%, 28–72%, and 25–42% increased grain yield compared to wild-type controls under stress conditions, and the grain yield of the root-specific overexpressors tended to be higher than that of the whole-plant overexpressor. Additionally, the transgenic rice showed similar or higher grain yields even in normal growth conditions compared to wild-type control plants. Molecular mechanisms underlying the NAC-dependent root growth and stress tolerance remain unknown, but it is expected that the increased root diameter improves drought tolerance by affecting root penetration through soil [20]. These findings indicated that promoting root growth improves tolerance to abiotic stress and rice productivity. This finding is supported by studies using *OsMADS25* that regulates root elongation and formation in rice by controlling the auxin response [82,98]. In this study, the knock-down mutant of *OsMADS25* with reduced root growth exhibited high sensitivity to salinity and oxidative stress, while transgenic rice overexpressing *OsMADS25* displayed enhanced tolerance to the abiotic stress [98].

In addition, a study of *DEEPER ROOTING 1* (*DRO1*) showed that modulation of the root system architecture affects rice yield under drought conditions [99]. In this study, they identified *DRO1* that regulates root architecture in rice. Since expression of *DRO1* is dramatically regulated by auxin, it is likely that auxin is deeply involved in the regulation of root architecture in rice. The angle of root growth was increased by *DRO1*, and consequently, roots of the rice with higher expression of *DRO1* grew in a more downward direction [99]. Furthermore, introduction of *DRO1* into the rice with a shallow-rooting system improved drought tolerance by promoting downward root growth, leading to enhanced drought tolerance and high grain yield under drought stress compared to the control plants. Additionally, the modulation of root system architecture by *DRO1* did not affect grain yield under normal growth conditions. Collectively, these suggest that modulation of root growth or architecture is a good strategy to develop stress-tolerant rice with no or minimal growth–defense trade-offs.

Root-specific lignification also affects stress tolerance in rice [100]. Lignin is hydrophobic and a key component of plant secondary cell walls, where it inhibits water loss from plant tissues [101]. Lignin biosynthesis is deeply involved in stress tolerance; for example, under drought conditions, maize drought-tolerant inbred lines exhibited increased accumulation of lignin compared with drought-sensitive lines [102]. Lee et al. (2016) revealed that *OsERF71* promoted lignin biosynthesis in rice by directly activating the expression of lignin biosynthesis genes such as *CINNAMOYL-COENZYME A REDUCTASE 1 (OsCCR1)*, and root-specific lignification by *OsERF71* promoted drought tolerance and increased grain yield up to 23–32% under drought conditions [100]. The role of lignin in rice stress tolerance is also supported by a recent study of *OsTF1L*, a rice *HD-Zip* transcription factor that regulates lignin biosynthesis [103]. 

## 5. Future Perspectives

Evolution of roots allowed land plants to survive and colonize the terrestrial environment [104]. Now, modulation of root growth could improve plant growth and tolerance to abiotic stress, and optimization or modulation of root development may enable the next Green Revolution [16]. The function of roots relies on the development of a variety of root tissues with specialized functions. For example, root hairs take up water and nutrients, the endodermis selectively transports nutrients, the vasculature is for long-distance transport of water, nutrient and carbon assimilates, and the aerenchyma functions in gas exchange. This suggests that modulation of root tissue development expands the possible approaches for the development of rice with improved yield and tolerance. However, despite significant progress in our understanding of rice root development, most developmental mechanisms underlying these processes remain largely unknown. Further molecular and genetic studies for the identification and characterization of genetic components involved in rice root development will provide important clues for the development of rice with higher yield and stress tolerance.
